# Effect of Mo and B on Microstructure and Impact Toughness of Coarse Grain Heat-Affected Zone in Low-Carbon V-Ti-N Micro-Alloyed Steel

**DOI:** 10.3390/ma18071667

**Published:** 2025-04-04

**Authors:** Mingliang Qiao, Huibing Fan, Shibiao Wang, Yixin Huang, Qingfeng Wang, Riping Liu

**Affiliations:** 1 State Key Laboratory of Metastable Materials Science and Technology, Yanshan University, Qinhuangdao 066004, China; 2 Nanjing Iron & Steel Co., Ltd., Nanjing 211500, China

**Keywords:** low-carbon micro-alloyed steel, CGHAZ, microstructures, impact toughness

## Abstract

This study investigates the effects of molybdenum (Mo) and boron (B) on the microstructures and impact properties in the coarse grain heat-affected zone (CGHAZ) of a low-carbon V-Ti-N steel. The results demonstrate that, at a heat input of up to 75 kJ/cm, the addition of Mo alters the microstructure of the CGHAZ, transforming it from a polygonal ferrite (PF) + degraded pearlite (DP) composition to a granular bainite (GB) + a small amount of acicular ferrite (AF). This transformation increases the impact energy from 20 J to 35 J. Furthermore, with the Mo/B composite addition, the CGHAZ microstructure was refined due to the formation of a large number of acicular ferrites, and the mean equivalent diameter (MED_MTA≥15°_) decreased from 7.9 μm to 4.2 μm. Consequently, the impact toughness of the CGHAZ increased from 35 J to 111 J. The correlation between the Mo/B elements, microstructure and impact toughness was investigated in detail. The findings of this study have the potential to generate novel ideas for the advancement of steel materials in the context of high heat input welding.

## 1. Introduction

With the increasing size of steel structural parts, to improve the manufacturing efficiency of steel structures, high heat input welding steel has recently been rapidly developed and achieved gratifying results [1,2,3]. However, low-carbon micro-alloyed high-strength steel for high heat input has not yet made a breakthrough. Under the condition of high heat input, the primary austenite grains in the CGHAZ are coarsened seriously, resulting in the appearance of coarse grain boundary ferrite, side lath ferrite and bainite in the CGHAZ of low-carbon high-strength steel, and lead to poor impact toughness of the CGHAZ [1,4,5]. The key to develop high-strength micro-alloyed steel with good weldability is to eliminate those coarse microstructures and refine the microstructure of the CGHAZ [6].

There’s plenty of evidence that TiN nanoparticles can exist stably or dissolve a little in the temperature range of 1200–1350 °C, so as to effectively nail the grain boundaries of primary austenite grains and inhibit the growth of primary austenite grains [7]. In addition, titanium, vanadium, nitrogen and carbon in steel can form VN, V(C,N) and (Ti,V)(C,N) particles, which promote non-uniform nuclear ferrite, thus achieving grain refinement [8,9,10]. Therefore, V-Ti-N micro-alloyed steel has received widespread attention as a new type of high heat input welding steel. Zhang et al. [6] added only 0.0064 wt.% N to V-Ti steel, which can also effectively promote the formation of intracrystalline ferrite. The N content in a wide range of 0.009–0.019 wt.% and the (V,Ti)(C,N) particles in the CGHAZ with a heat input of 100 kJ/cm have the effect of stably promoting IGF nucleation, which makes the CGHAZ have good low-temperature toughness [11]. However, the microstructural refinement in the CGHAZ for high heat input welding of the low-carbon V-Ti-N steel is still insufficient, owing to the formation of IGPF at a higher temperature. Accordingly, the strength/toughness combination of this steel is inadequate.

Fortunately, by decreasing the phase transition temperature, the morphology of intergranular ferrite (IGF) changes from polygon to star-shaped acicular ferrite to achieve the refinement of the microstructures [12]. Adding a certain amount of Mo element could lower the phase transition temperature for the IGF and promote the IGAF [13]. However, the coarsened GBPF and SPF generated at the PAG boundaries worsen the toughness of the CGHAZ still existing [14,15,16]. In order to inhibit the development of coarse microstructure at the grain boundaries, the introduction of B element in steel may be a good choice. During the cooling process of the CGHAZ, B will segregate at the grain boundaries to reduce the phase transition temperature and inhibit the growth of coarse microstructures [17,18]. Moreover, B can combine with N to form BN particles, and BN particles promote the acicular ferrite nucleus in the grain, which further refines the CGHAZ structure. Mo/B composite addition is expected to develop high-heat input high-strength structural steel. However, the effect of Mo/B on the microstructure and mechanical properties of the CGHAZ is rarely reported.

In this study, the influence of Mo and B on the microstructure and impact toughness of the simulated CGHAZ of a low-carbon micro-alloy steel was carefully investigated. In the laboratory, experimental steels with varying compositions were prepared for a CGHAZ simulations experiment. The relationships between the Mo and B elements, microstructure, and impact toughness in the CGHAZ were examined using simulated samples.

## 2. Materials and Methods

Three micro-alloyed steels with differing chemical compositions were manufactured via a 50 kg vacuum furnace in order to ascertain the effect of Mo and B on the microstructure and impact toughness of the CGHAZ. The specific chemical components of the experimental steels were determined by Spectrometer HK-780(Nanjing Huake Analytical Instrument Co., Nanjing, China), and the results are shown in Table 1. After 2 h of automatic processing at 1200 °C, the billets were subjected to a controlled rolling process, followed by controlled cooling. The initial rough rolling stage was conducted at a temperature of 1100 °C, resulting in a cumulative reduction of 80 mm. This was followed by a finishing rolling stage that commenced at 950 °C and concluded at 850 °C, achieving a cumulative reduction of 52 mm. Following the attainment of a final thickness of 18 mm, the steels were subjected to water cooling at a rate of 15 °C/s from 815 °C to 400 °C, after which they were then air-cooled to ambient temperature.

**Figure 1:**
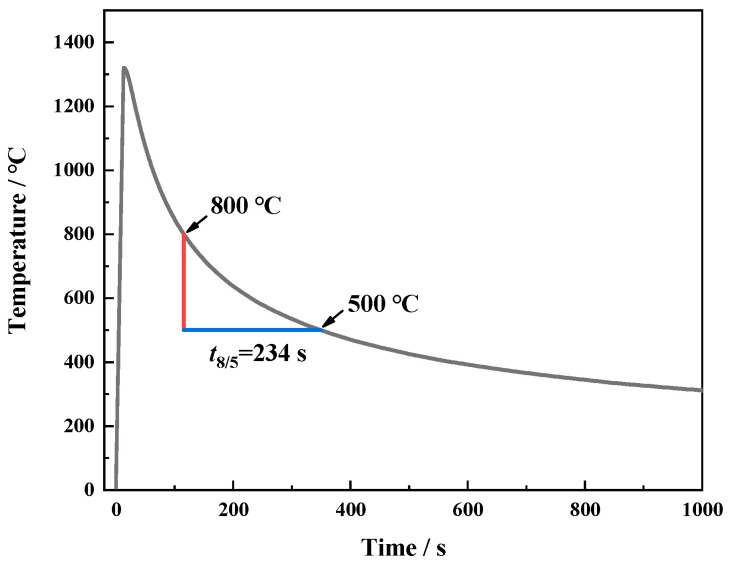
Thermal simulation process curve.

Gleeble-3800(Dynamic Systems Inc., New York, NY, USA) was used to simulate the thermal cycle experienced by the CGHAZ of a welding 18 mm thick steel with 75 kJ/cm. Figure 1 shows the specific thermal simulation curve. The round bar samples (Φ6 mm × 80 mm) and cuboid samples (10.5 mm × 10.5 mm × 80 mm) cut from the sheet along the longitudinal direction were heated to 1320 °C at a speed of 100 °C/s and held for 1 s, then cooled to 200 °C along the thermal cycle curve, and finally, they were air-cooled to ambient temperature. The round bar specimens were used to determine the thermal expansion curves and CGHAZ microstructure.

The simulated sample (10.5 mm × 10.5 mm × 80 mm) was machined into a Charpy V-notch sample (10 mm × 10 mm × 55 mm). The V-notch was fabricated in the middle of the sample where the thermocouple was located. To analyze the impact fracture behavior of the CGHAZ, the displacement–load curves of the simulated impact specimens were measured at −40 °C using an oscillographic impact tester (PSW1000, Guangdong Yueh-Lian Instrument Co., Dongguan, China). The reported impact energy in this paper was the average of three parallel impact samples.

Metallographic samples were obtained from the simulated samples along the cross-section of the thermocouple position. Following grinding, polishing and etching with a 3% nitrate ethanol solution, the samples were examined using an Olympus BX51M optical microscope (Olympus, Aizu, Japan). Subsequent to this, the metallographic samples were repolished, and then electropolished in a solution of 10% perchloric acid, 5% glycerol and 85% alcohol (polishing voltage: 18 V; current: 0.5~1 mA). Following this, the samples were characterized using an Electron Backscatter Diffraction (EBSD) system on a SU-5000 scanning electron microscope (SEM SU5000, HITACHI, Tokyo, Japan), with a scan step of 0.2 μm. This was followed by the characterization of the misorientation distribution. The average equivalent diameter (MED) of the microstructures with misorientation angles of 15° was determined using EBSD. The reported MED was the mean size of at least 300 grains measured. The microstructural features were then investigated using a high-resolution transmission electron microscope (TEM, Japan Electronics optics Corporation, Tokyo Prefecture, Japan). The impact fracture morphologies of the simulated samples were observed and characterized using a scanning electron microscope (SEM). The secondary crack extension path was examined using an SEM and an EBSD.

## 3. Results

### 3.1. Microstructure of the Simulated Samples

**Figure 2:**
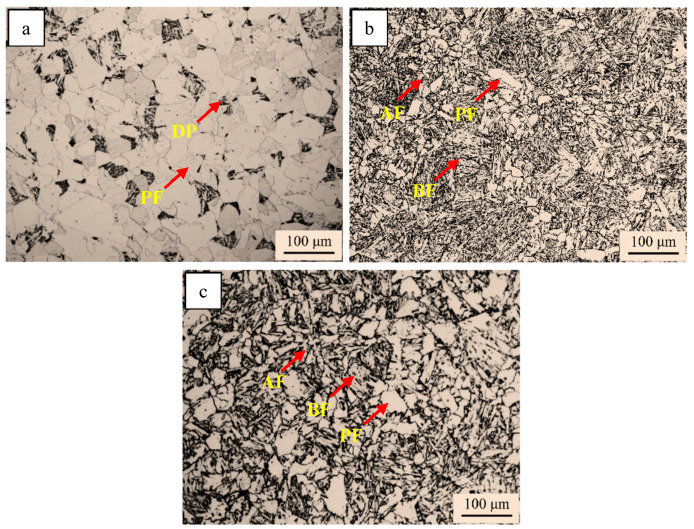
Optical micrographs of simulated samples of (**a**) steel A; (**b**) steel B; (**c**) steel C.

The optical micrographs of the simulated samples with different chemicals are shown in Figure 2. In addition, the quantification results of the microstructure in the simulated samples are provided in Table 1. The simulated steel A sample was composed of abundant PF and DP (Figure 2a). Compared with the simulated steel A sample, the DP disappeared, the PF reduced, and AF and GB appeared in the steel B simulated sample (Figure 2b). The microstructure of the steel C sample consisted of a mass of PF and AF and a little of GB.

**Figure 3:**
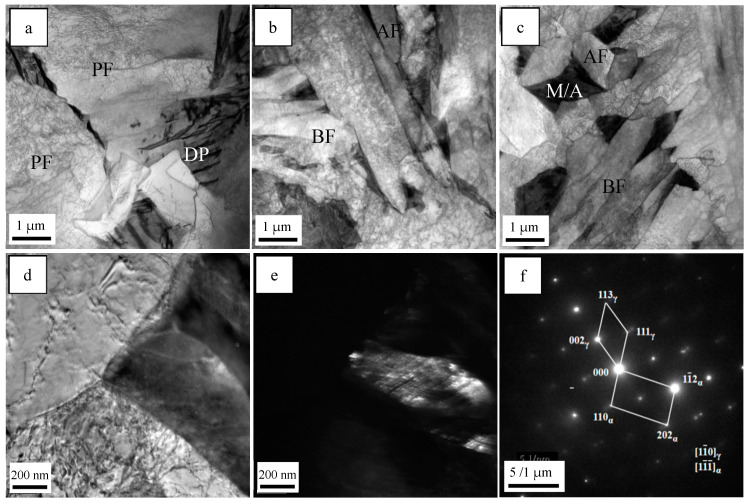
TEM micrographs of simulated samples of (**a**) steel A; (**b**) steel B; (**c**) steel C, (**d**) bright field, (**e**) dark field, (**f**) the selected area diffraction pattern.

The TEM micrographs of the simulated samples are provided in Figure 3. There were DP and PF with low-density dislocations in the simulated sample of steel A, while the microstructure of the simulated steel B sample was mainly composed of AF, BF and M/A constituents with the shape of islands. The microstructure of the simulated steel C sample consisted of fine AF, a finer BF and some M/A constituents. Moreover, the M/A constituent was confirmed using the bright field image (Figure 3d), dark field image (Figure 3e) and electron diffraction image (Figure 3f).

**Figure 4:**
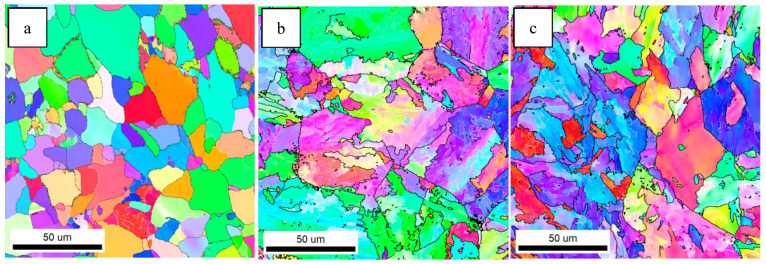
Inverse polarity diagrams of simulated samples of (**a**) steel A; (**b**) steel B; (**c**) steel C.

Previous studies have shown that high-angle grain boundaries (HAGBs) can inhibit crack propagation and improve the impact toughness of steel. The crystallographic information of the test steels was characterized by EBSD. Figure 4 shows the inverse polarity diagrams of the simulated samples. According to the misorientation tolerance angle (MTA) between the adjacent grains, the grain boundaries are classified as low-angle grain boundaries (LAGBs) (MTA < 15°) and HAGBs (MTA ≥ 15°), which are distinguished by white and black lines, respectively.

**Figure 5:**
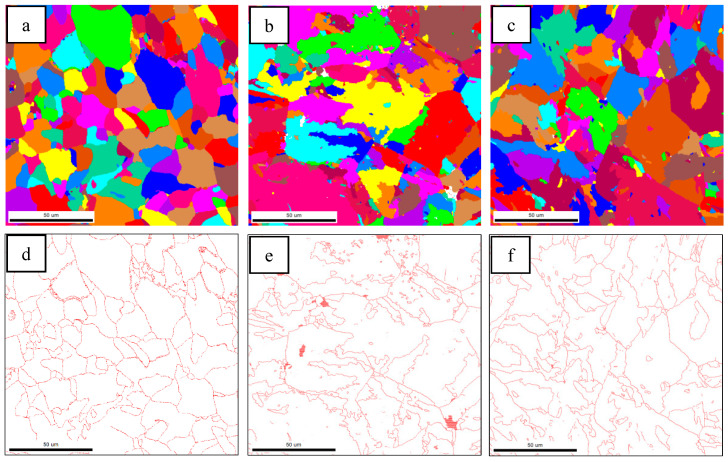
EBSD unique grain color figure and corresponding HAGB diagram for steel A (**a**,**d**), steel B (**b**,**e**) and Steel C (**c**,**f**).

HAGBs could force the cracks to change their propagation paths and hinder the expansion of the cracks effectively [19,20]. Figure 4 indicates that the grain boundaries between the AF and GB are HAGBs, and the same goes for the grain boundaries between PF. The unique color grain and corresponding HAGB diagram were drawn by EBSD, as shown in Figure 5. The order of effective grain sizes defined at 15° from coarse to fine was test steel A, test steel B and test steel C, and the mean grain size of test steel A, test steel B and test steel C was 10.1 μm, 7.9 μm and 4.2 μm, respectively. The grain refinement can be attributed to the increase in AF content.

### 3.2. Impact Toughness of the Simulated Specimens

**Figure 6:**
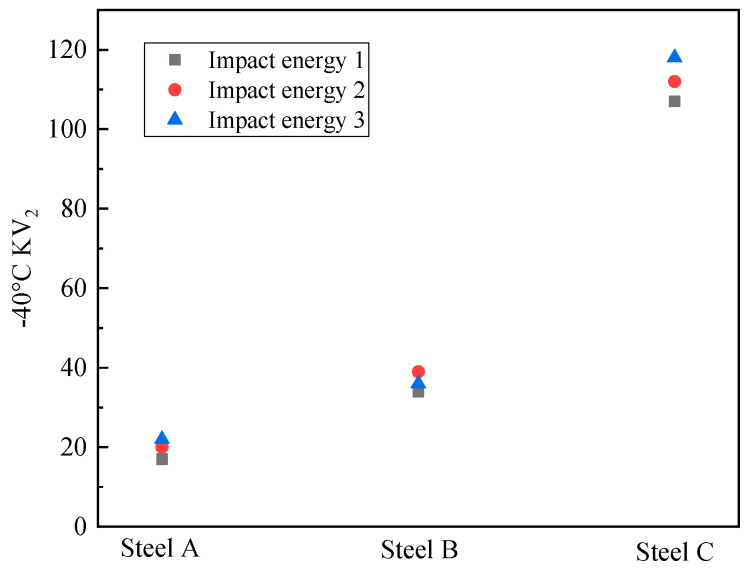
Pendulum impact results of simulated samples.

**Figure 7:**
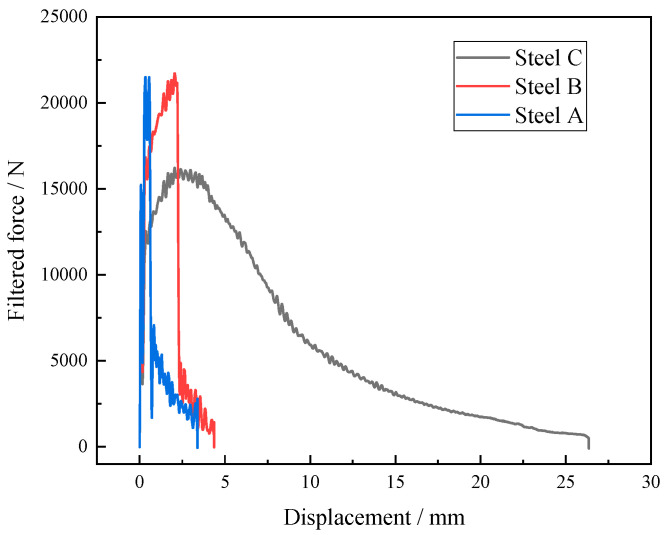
Impact load–deflection curves for simulated samples at −40 °C.

The impact test results of the simulated samples at −40 °C are exhibited in Figure 6. The impact energies of the steel A, steel B and steel C samples were 20 J, 35 J and 111 J, respectively. The impact toughness of the CGHAZ for the test steels was considerably improved when Mo and B were added at the same time. Figure 7 presents the force–displacement curves of the simulated samples at temperatures of −40 °C. The crack initiation energy (E_i_) and crack propagation energy (E_j_) were obtained from the areas enclosed to the left and right of the highest point, respectively. The total impact energy (E_t_), E_i_, and E_j_ are shown in Table 2.

**Figure 8:**
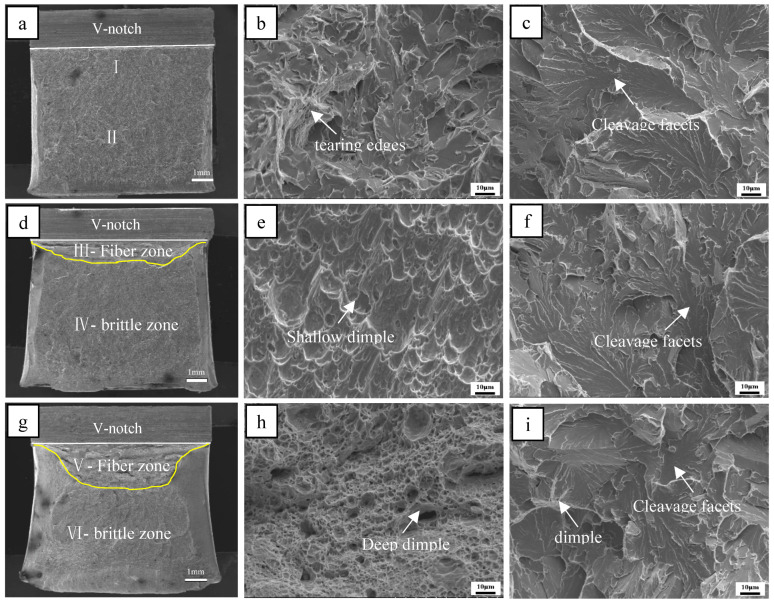
Impact fracture morphology for steel A sample (**a**), steel B sample (**d**) and steel C sample (**h**) at −40 °C. Characterization ((**b**)/(**c**), (**e**)/(**f**) and (**h**)/(**i**)) of positions marked as I/II in steel A sample (**a**), as III/IV in steel B sample (**d**) and as V/VI in steel C sample (**g**).

The morphology features of the fracture surfaces of the steel A, steel B and steel C samples at −40 are shown in Figure 8. Figure 8a,d,g present the macroscopic fracture morphology of the steel A, steel B and steel C samples, respectively. There is little fiber zone in the steel A sample, while the fiber area percentage of the steel B sample is about 9%. Steel sample C presents a maximum fiber area of 36%.

Figure 8b,c show the morphology of the regions near the v-notch (I) and radiation region (II) in Figure 8a. The fracture plane near the V-notch is mainly composed of small cleavage planes and some tearing edges. The cleavage region in sample A is mainly a large flat cleavage plane. The fiber zone of the B sample consists of flat dimples, and the radiation region is made up of large flat cleavage planes, as shown in Figure 8e,f, respectively. Figure 8h,i show the morphology of the fiber region (V) and the radiation region (VI) in Figure 8g. The fiber zone of the C sample consists of deep dimples, and the radiation region is made up of small cleavage planes. For the −40 °C impact samples, the area of the fiber zone increased and the area of the radiation zone decreased when the microstructure changed from DP + PF to AF + GB + M/A. The dimples in the fiber region became deeper and the cleavage plane in the extended region decreased.

## 4. Discussion

### 4.1. The Effect of Mo and B on Microstructure

**Figure 9:**
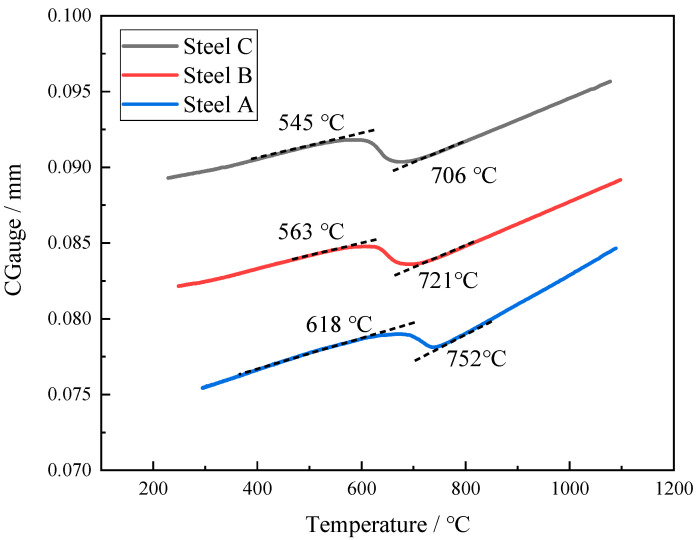
The dilatation curves and the Ar3 points for the simulated samples.

The morphology of ferrite is related to the phase transition temperature. PF is formed at a high temperature, and acicular ferrite and lath ferrite are formed at relatively low temperatures [12]. Figure 9 shows the expansion curves and phase transition temperatures of the three test steels during cooling. The phase Ar3 of test steel A was 752 °C, and the phase transition temperature was relatively high. When cooling to 650 °C, the phase transition was basically completed before entering the acicular ferrite transformation range for steel A. PF is a diffusion phase transition transformation controlled by the diffusion and migration of solute atoms near the phase interface [21,22]. The carbon atoms are fully diffused at a relatively high temperature and the growth rate of the ferrite is independent of the grain boundary orientation. Therefore, the main microstructures consist of PF in the A steel. When large-sized PF is formed at a high temperature, the ferrite emits more carbon, and the carbon elements gather into the untransformed austenite. With further reduction in temperature, the austenite with higher carbon content is transformed into large-sized degenerate pearlite. As a result, the microstructure of test steel A is PF and DP.

Figure 9 shows that adding a certain amount of Mo to the steel can significantly decline the Ar3. The transition temperature of the test steel was reduced from 752 °C to 700 °C. The diffusion rate of the atoms decreased gradually with the temperature. In addition, the addition of Mo causes the diffusion coefficient of carbon to decrease [13,23]. As a result, the non-diffusion phase transformation was more likely to occur at lower temperatures rather than the diffusion-controlled phase transitions in steel B. The PF transformation was inhibited due to the reduced diffusion rate of the carbon atoms. Therefore, there was only a small amount of PF in test steel B. The transformation of austenite mainly occurs between 545 °C and 650 °C, which is the medium-temperature transition zone [24,25]. The bainite transformation occurs and the amount of granular bainite is generated.

**Figure 10:**
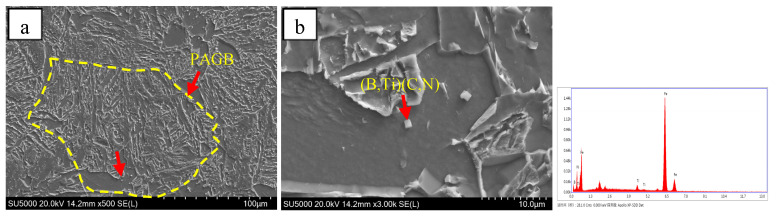
SEM of grain boundary ferrite nucleation on (B,Ti)(C,N) precipitated particles in test steel C (**a**), The enlarged view of site indicated by the arrow in Figure (**b**), and corresponding EDS of (B,Ti)(C,N) particles.

**Figure 11:**
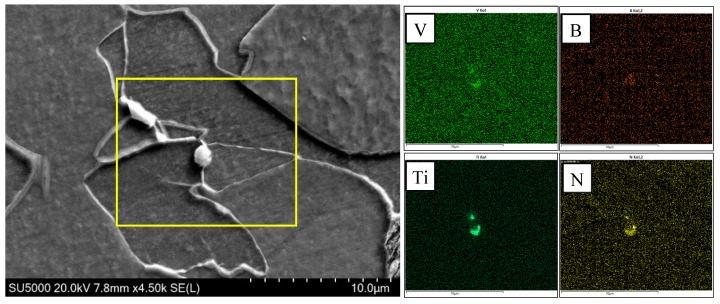
SEM of ferrite nucleation on (B,Ti)(C,N) precipitateds particle and element distribution of (B,Ti)(C,N) particles.

**Figure 12:**
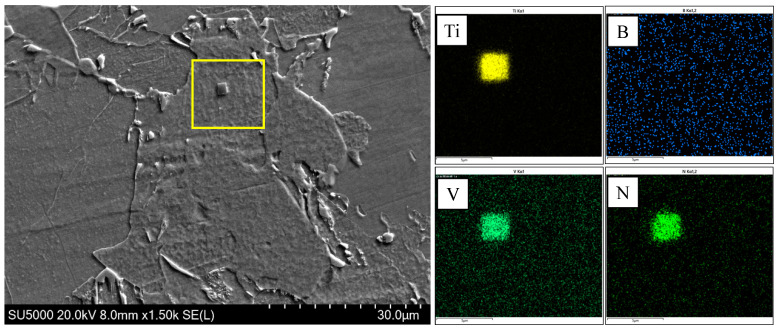
SEM of ferrite nucleation on (V,Ti)(C,N) precipitated particles and element distribution of (V,Ti)(C,N) particles.

Figure 9 indicates that there was little difference in the transition temperature interval between test steel B and test steel C. However, there was a large amount of AF and only a small amount of GB in steel C, which could be attributed to the element B. During the cooling process, B tends to segregate at the original austenite grain boundary [26,27,28,29]; the B content near the grain boundary increases and combines with the N element to precipitate on the Ti(C,N) to form (B,Ti)(C,N), and the (B,Ti)(C,N) particles at the grain boundary provide nucleation sites for the ferritic nucleus, so that more ferrite is preferentially formed at the grain boundary, as shown in Figure 10. The formation of ferrite preferentially occupies the austenite grain boundary and thus inhibits the transformation of bainite. At the same time, there are numbers of precipitated (V,Ti)(C,N) and (B,Ti)(C,N) particles in the austenite grain to provide nucleating sites for the ferrite (Figure 11 and Figure 12), which can promote the formation of intragranular ferrite. For steel B, GB firstly nucleated at the grain boundaries instead of the AF nucleated on the precipitates, and the GB grew rapidly into the grain [30], which inhibited the AF transformation. Therefore, GB was the dominant microstructure in steel B.

After the completion of the ferrite transformation, part of the carbon-rich austenite was retained. During the subsequent cooling, part of the carbon-rich austenite was transformed into martensite, and part of the residual austenite coexisted with the martensite, namely the M/A constituent.

### 4.2. Effect of Ferrite Morphology on Impact Toughness

**Figure 13:**
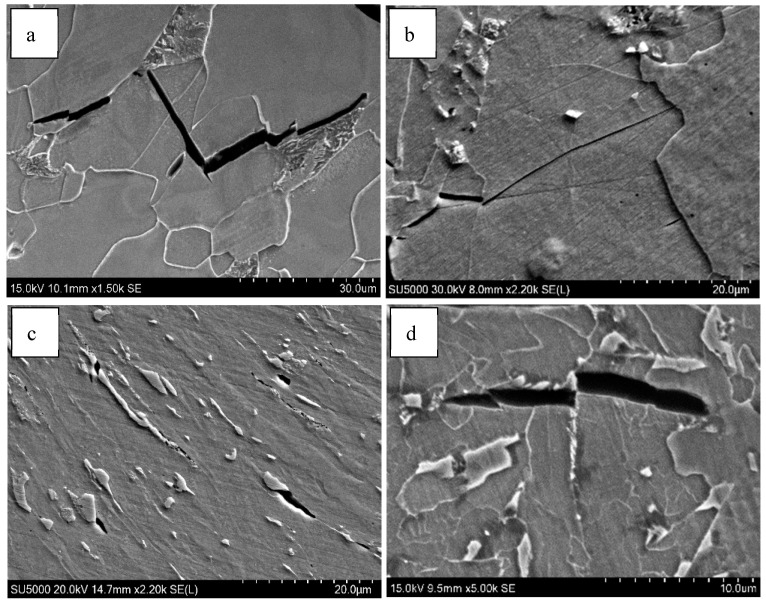
SEM observations of secondary crack in brittle zones of steel A (**a**,**b**) and steel C (**c**,**d**) samples.

Impact energy is generally divided into two parts: crack initiation energy and crack propagation energy. Figure 13 shows the morphology of the crack initiation and crack propagation. The crack initiates at the hard-phase DP and M/A constituents, as shown in Figure 13a,c.

In general, crack initiation usually goes through the following process [31]. Under the impact load, the soft phase and the hard phase in the microstructure first undergo cooperative elastic deformation, followed by the plastic deformation of the soft phase, resulting in the local microscopic strain concentration occurring at the interface of the hard phase or/and the soft phase. The subsequent continuous increase in strain may lead to microcracks or/and micropores in these regions. KAM plots can be used to estimate local micro-strain levels [32].

**Figure 14:**
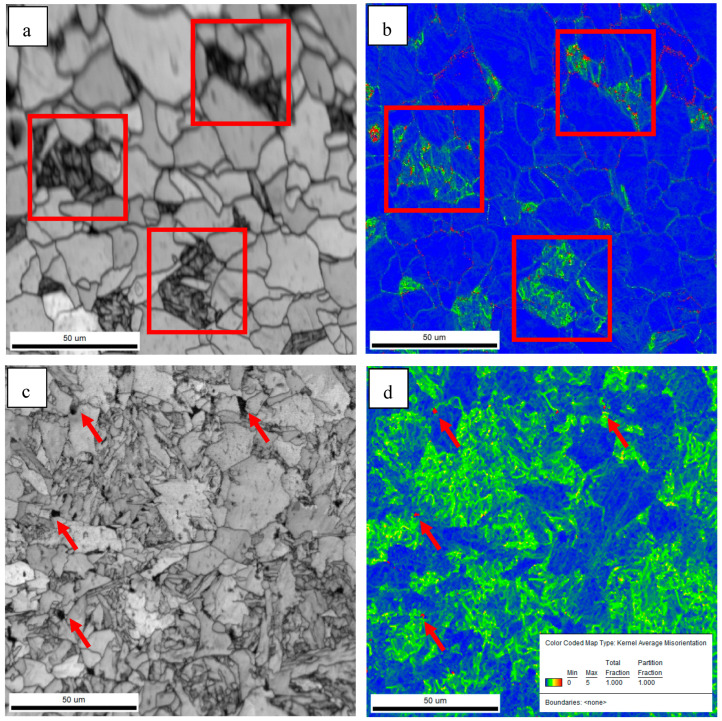
Band contrast maps and KAMs of steel A sample (**a**,**b**) and steel C sample (**c**,**d**).

**Figure 15:**
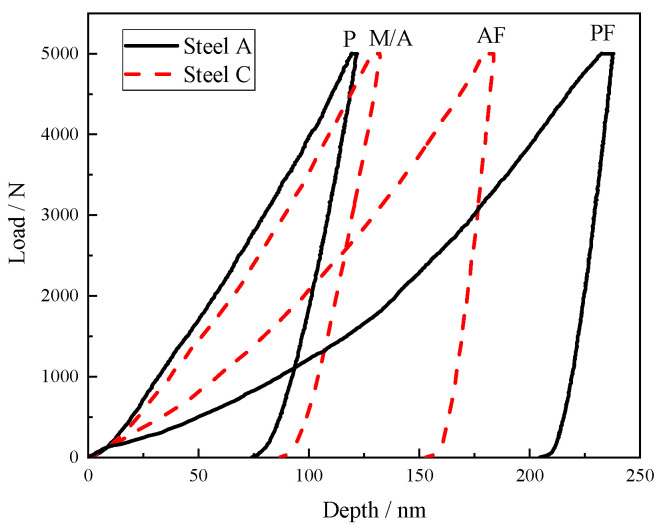
Typical load–depth curves of P and M/A constituent and matrix nanoindentations.

The thermal expansion coefficient of the M/A constituents and DP is different from that of the matrix. During the cooling process, stress will be formed inside and near the contact surface of M/A and DP, resulting in stress concentration as shown in Figure 14. At the same time, it is generally believed that the soft substrate around the hard phase usually happens with the initial plastic deformation under the impact load, generating a considerable local stress concentration and then causing crack [8]. The hardness between the pearlite and M/A constituents and the adjacent ferrite matrix was evaluated by nano indentation test. The results are shown in Figure 15 and Table 3. The hardness of the pearlite and M/A constituents is quite different from that of the matrix. The pearlite and M/A constituents are hard-phase with higher strength, so the indentation is shallow, while AF and PF are soft-phase, so the indentation is deep. Under the same load force, the indentation depth of the soft and hard phases is different, indicating that the deformation is different. As a result, it is easy to initiate cracks at the pearlite and M/A constituents during impact. The hardness of the pearlite and M/A constituents is close; therefore, there is little difference in hardness between them and the matrix, while the size of the pearlite is much larger than that of the M/A constituent. The larger the hard-phase size is, the less cooperative deformation is likely to occur [33]. The size of the pearlite is much larger than that of the M/A constituent. As a result, the pearlite is more likely to initiate cracks than the M/A constituent, resulting in a lower crack initiation energy. The size of the M/A constituent is much smaller than the DP, small micro-voids appear at the interface between the M/A and the matrix without forming cracks, and large rheology occurs near the fracture. Therefore, the crack initiation energy of the test steel C sample is higher.

**Figure 16:**
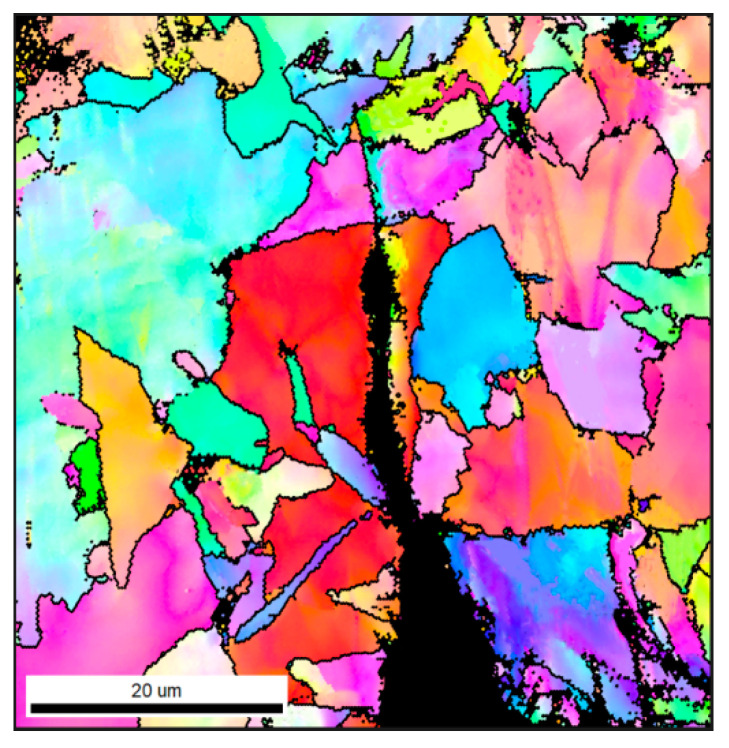
IPF of secondary cracks of simulated steel C sample.

In the Figure 16, the black line represents the grain boundary with an orientation angle higher than 15° (high-angle grain boundary). The crack goes straight through in the grain, indicating that the low-angle grain boundary cannot hinder the crack propagation. Passing through the high-angle grain boundary, the cracks turn and narrowed in width, and finally stop at the HAGB. The HAGB can hinder crack propagation [34,35]. Grain refinement occurs when the microstructure of the simulated sample changes from GB to AF, and the effective grain size decreases from 7.9 μm to 4.2 μm. Therefore, the steel C sample has a stronger ability to hinder the crack propagation than the steel B sample, and more energy is consumed when the crack propagates in the acicular ferrite sample. Therefore, the crack propagation energy of the acicular ferrite sample is higher than that of the massive ferrite sample.

**Figure 17:**
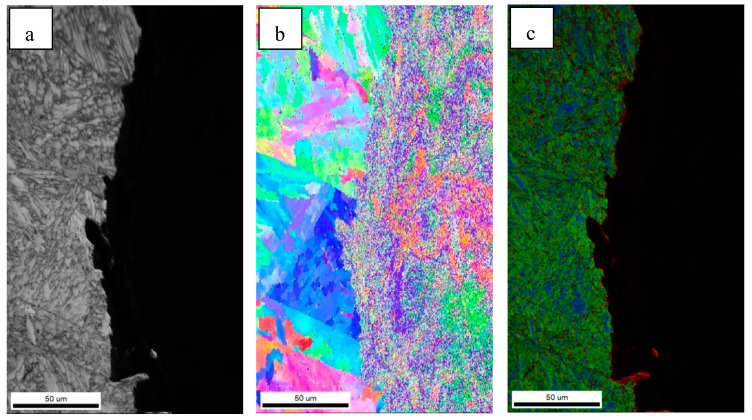
EBSD characterization of main crack of steel B simulated sample: (**a**) IQ, (**b**) IPF, (**c**) KAM.

**Figure 18:**
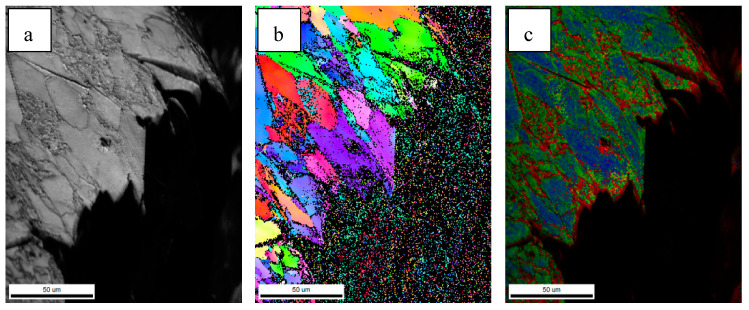
EBSD characterization of main crack of steel C simulated sample: (**a**) IQ, (**b**) IPF, (**c**) KAM.

Figure 17 and Figure 18 show the EBSD diagram of the main fracture in the crack expansion zone of the impact specimens of steel B and test steel C, respectively. Figure 17 shows that the deformation of bainite during the impact is small, which is consistent with the results of the main fracture in Figure 8d. Little deformation occurred in the adjacent fracture surface (Figure 17c). Figure 18 shows that the structure of the PF and AF possesses an obvious deformation near the fracture, which is consistent with the fracture in Figure 8h. The above results show that steel C absorbs more potential energy and transforms it into plastic deformation energy during the impact process. The appearance of the acicular ferrite refined the grains of the CGHAZ. At the same time, acicular ferrite possesses good plastic deformation ability, which made test steel C have good impact toughness.

## 5. Conclusions

An investigation was conducted into the microstructural evolution, impact properties and fracture mechanism of a low-C Mo-V-Ti-B steel with different compositions, utilizing a heat input of 75 kJ/cm. The primary conclusions of this study can be summarized as follows:

1. Adding 0.28% Mo element to the V-Ti micro-alloy steel significantly reduces the Ar3 from 752 °C to 721 °C and delays the transformation of austenite, and the microstructure of the CGHAZ changes from PF + DP to GB + a small amount of AF.
2. When Mo and B elements are added together, B segregated at the grain boundary and combined with N in the steel and precipitates on the Ti(C,N) particles to form composite (B,Ti)(C,N) particles. The (B,Ti)(C,N) particles at the grain boundary act as nucleation sites of ferrite, and the ferritic transformation occurs preferentially at the austenite grain boundary. The formation of ferrite occupies the nucleation position of bainite and inhibits the transformation of GB, thus creating the conditions for the nucleation and growth of ferrite on the precipitated particles in the austenite grain. The microstructure of the CGHAZ changes from GB + a little AF to a small amount of PF + a large amount of AF, which makes the microstructure of the CGHAZ refined, and the mean grain size decreases from 10.1 μm to 4.2 μm.
3. The impact toughness of the CGHAZ of the test steel was significantly improved from 20 J to 111 J by adding Mo and B. Meanwhile, the fracture behavior changed from brittle fracture to ductile fracture, which could be attributed to a decrease in the size of the hard constituents and the microstructure refinement and the good deformation ability of acicular ferrite.

## Figures and Tables

**Table 1:** The chemical composition of the experimental steels, wt.%.

Steel	C	Si	Mn	S	P	Mo	V	Ti	N	B
A	0.077	0.20	1.50	0.006	0.007	-	0.060	0.015	0.0091	-
B	0.065	0.293	1.55	0.002	0.004	0.28	0.067	0.017	0.0097	-
C	0.063	0.274	1.55	0.001	0.004	0.28	0.066	0.016	0.0110	0.0011

**Table 2:** Summary statistics of microstructure identification and quantification.

Experimental Steels	Phase Composition Fraction	E_t_/J	E_i_/J	E_j_/J
Steel A	78% PF + 22% DP	20	9	11
Steel B	23% PF + 13% AF + 64% GB	35	29	6
Steel C	71% AF + 18% GB + 11% PF	111	23	88

**Table 3:** Summary of nanoindentation hardness of hard phase and ferrite matrix.

Simple	Matrix/GPa	Hard Phase/GPa	Difference/GPa
Steel A	3.6	8.7	4.1
Steel C	3.4	6.9	3.5

## Data Availability

The original contributions presented in the study are included in the article, further inquiries can be directed to the corresponding author.

## References

[1] Yang Y.L., Jia X., Ma Y.X., Wang P., Zhu F.X., Yang H.F., Wang C., Wang S.G. (2022). Effect of Nb on Microstructure and Mechanical Properties between Base Metal and High Heat Input Coarse-Grain HAZ in a Ti-Deoxidized Low Carbon High Strength Steel. J. Mater. Res. Technol..

[2] Li T.T., Yang J., Zhang Y.H., Zhang Y.Q., Chen Y.L., Xu L.Y., Li R.B. (2024). Inclusions and Microstructures in Coarse-Grained Heat-Affected Zone of Al–Ti–Ca Deoxidized Shipbuilding Steels with Different *Al* Contents after High-Heat Input Welding. J. Mater. Res. Technol..

[3] Shen Y., Leng J., Wang C. (2019). On the Heterogeneous Microstructure Development in the Welded Joint of 12MnNiVR Pressure Vessel Steel Subjected to High Heat Input Electrogas Welding. J. Mater. Sci. Technol..

[4] Hu J., Du L.X., Wang J.J., Gao C.R. (2013). Effect of Welding Heat Input on Microstructures and Toughness in Simulated CGHAZ of V–N High Strength Steel. Mater. Sci. Eng. A.

[5] Zhang J., Xin W.B., Ge Z.W., Luo G.P., Peng J. (2023). Effect of High Heat Input Welding on the Microstructures, Precipitates and Mechanical Properties in the Simulated Coarse Grained Heat Affected Zone of a Low Carbon Nb-V-Ti-N Microalloyed Steel. Mater. Charact..

[6] Qiao M., Fan H., Shi G.H., Wang L.P., Wang Q.M., Wang Q.F., Liu R.P. (2021). Effect of Welding Heat Input on Microstructure and Impact Toughness in the Simulated CGHAZ of Low Carbon Mo-V-Ti-N-B Steel. Metals.

[7] Shi Z., Wang R., Su H., Chai F., Wang Q., Yang C. (2016). Effect of Nitrogen Content on the Second Phase Particles in V–Ti Microalloyed Shipbuilding Steel during Weld Thermal Cycling. Mater. Des..

[8] Fan H.B., Shi G.H., Peng T., Wang Q.M., Wang L.P., Wang Q.F., Zhang F.C. (2021). N-Induced Microstructure Refinement and Toughness Improvement in the Coarse Grain Heat-Affected Zone of a Low Carbon Mo–V–Ti–B Steel Subjected to a High Heat Input Welding Thermal Cycle. Mater. Sci. Eng. A.

[9] Garcia-Mateo C., Capdevila C., Caballero F.G., de Andrés C.G. (2008). Influence of V Precipitates on Acicular Ferrite Transformation Part 1: The Role of Nitrogen. ISIJ Int..

[10] Hu B., Shi G.H., Wang Q.M., Zhao L.Y., Fan H.B., Tang Y.C., Wang W., Wang Q.F., Liu R.P. (2023). Elucidating the Heat Input on CGHAZ Microstructure and Its Irregular Effect on Impact Toughness for a Novel V–N Microalloying Weathering Steel. J. Mater. Res. Technol..

[11] Shi Z.R., Yang C.F., Wang R.Z., Su H., Chai F., Chu J.F., Wang Q.F. (2016). Effect of Nitrogen on the Microstructures and Mechanical Properties in Simulated CGHAZ of Vanadium Microalloyed Steel Varied with Different Heat Inputs. Mater. Sci. Eng. A.

[12] Cheng L., Wu K.M. (2009). New Insights into Intragranular Ferrite in a Low-Carbon Low-Alloy Steel. Acta Mater..

[13] Kong J., Lin Z., Bin G., Pinghe L., Aihua W., Changsheng X. (2004). Influence of Mo Content on Microstructure and Mechanical Properties of High Strength Pipeline Steel. Mater. Des..

[14] Yamamoto K., Matsuda S., Haze T., Chijiiwa R., Mimura H. (1989). A Newly Developed Ti-Oxide Bearing Steel Having High HAZ Toughness. Residual and Unspecified Elements in Steel.

[15] Zafra A., Álvarez G., Belzunce J., Alegre J.M., Rodríguez C. (2021). Fracture Toughness of Coarse-Grain Heat Affected Zone of Quenched and Tempered CrMo Steels with Internal Hydrogen: Fracture Micromechanisms. Eng. Fract. Mech..

[16] Tweed J.H., Knott J.F. (1987). Micromechanisms of Failure in C-Mn Weld Metals. Acta Metall..

[17] Fujishiro Y., Hashimoto T., Ohtani H. (1988). Influence of Boron and Nitrogen Contents on Strength and Toughness of Controlled-Rolled Low Carbon-Boron Steel. Scr. Metall..

[18] Kim S., Kang Y., Lee C. (2013). Variation in Microstructures and Mechanical Properties in the Coarse-Grained Heat-Affected Zone of Low-Alloy Steel with Boron Content. Mater. Sci. Eng. A.

[19] Zhong Y., Xiao F., Zhang J., Shan Y., Wei W., Yang K. (2006). In Situ TEM Study of the Effect of M/A Films at Grain Boundaries on Crack Propagation in an Ultra-Fine Acicular Ferrite Pipeline Steel. Acta Mater..

[20] Pandey C., Mahapatra M.M., Kumar P., Thakre J., Saini N. (2018). Role of Evolving Microstructure on the Mechanical Behaviour of P92 Steel Welds Joint in As-Welded and Post Weld Heat Treated State. J. Mater. Process. Technol..

[21] Zhou X., Dong J., Liu Y., Liu C., Yu L., Huang Y., Li H. (2017). Austenite to Polygonal-Ferrite Transformation and Carbide Precipitation in High Strength Low Alloy Steel. Int. J. Mater. Res..

[22] Park K.-T., Hwang S.W., Ji J.H., Lee C.H. (2011). Inclusions Nucleating Intragranular Polygonal Ferrite and Acicular Ferrite in Low Alloyed Carbon Manganese Steel Welds. Met. Mater. Int..

[23] Liu J., Li Z., Yang G., Liu X., Du X., Shi W., Yang S. (2024). Effect of Mo addition on microstructure and mechanical properties of Cu-12.5Ni-5Sn alloy. J. Mater. Today Commun..

[24] Jung Y.-C., Kim S.-J., Ohmori Y. (1998). Morphology and Growth Process of Bainitic Ferrite in Steels. Met. Mater. Int..

[25] Takayama N., Miyamoto G., Furuhara T. (2012). Effects of Transformation Temperature on Variant Pairing of Bainitic Ferrite in Low Carbon Steel. Acta Mater..

[26] Li Y., Ponge D., Choi P., Raabe D. (2015). Atomic Scale Investigation of Non-Equilibrium Segregation of Boron in a Quenched Mo-Free Martensitic Steel. Ultramicroscopy.

[27] Takahashi J., Ishikawa K., Kawakami K., Fujioka M., Kubota N. (2017). Atomic-Scale Study on Segregation Behavior at Austenite Grain Boundaries in Boron- and Molybdenum-Added Steels. Acta Mater..

[28] Shi Z., Wang J., Chai X., Wang S., Chen G., Wang R. (2020). Effect of Boron on Intragranular Ferrite Nucleation Mechanism in Coarse Grain Heat-Affected Zone of High-Nitrogen Steel. Mater. Lett..

[29] Ilman M.N., Cochrane R.C., Evans G.M. (2013). Effect of Nitrogen and Boron on the Development of Acicular Ferrite iN Reheated C-Mn-Ti Steel Weld Metals. Weld. World.

[30] Xu Z. (2006). A Brief Introduction to Bainitic Transformation. Heat Treat..

[31] Tong M., Di X., Li C., Wang D. (2018). Toughening mechanism of inter-critical heat-affected zone in a 690 MPa grade rack plate steel. Mater. Charact..

[32] Zhao L., Wang Q., Shi G., Yang X., Qiao M., Wu J., Zhang F. (2022). In-depth understanding of the relationship between dislocation substructure and tensile properties in a low-carbon microalloyed steel. Mater. Sci. Eng. A.

[33] Tian D., Karjalainen L., Qian B., Chen X. (1997). Cleavage Fracture Model for Granular Bainite in Simulated Coarse-Grained Heat-Affected Zones of High-Strength Low-Alloyed Steels. JSME Int. J. Ser. A Solid Mech. Mater. Eng..

[34] Wang X., Wu Z., Zhang N., Wang C., Yuan G. (2024). An Improved Toughness Process for High-Temperature Hot-Rolled HSLA Steel via Inclusion-Induced Acicular Ferrite Nucleation. Mater. Lett..

[35] Lv S., Wu H.-H., Wang K., Wang S., Wu G., Gao J., Yang X.-S., Zhu J., Mao X. (2023). The Microstructure Evolution and Influence Factors of Acicular Ferrite in Low Alloy Steels. Comput. Mater. Sci..

